# Defibrillation threshold of internal cardioversion prior to ablation predicts atrial fibrillation recurrence

**DOI:** 10.1002/clc.23679

**Published:** 2021-06-23

**Authors:** Kohei Sawasaki, Yasuya Inden, Natsuko Hosoya, Masahiro Muto, Toyoaki Murohara

**Affiliations:** ^1^ Department of Cardiology Hamamatsu Medical Center Hamamatsu Japan; ^2^ Department of Cardiology Nagoya University Graduate School of Medicine Nagoya Japan

**Keywords:** atrial defibrillation threshold, catheter ablation, persistent atrial fibrillation

## Abstract

**Background:**

Many studies have reported the predictors of atrial fibrillation (AF) recurrence after persistent AF (peAF) ablation. However, the correlation between the atrial defibrillation threshold (DFT) for internal cardioversion (IC) and AF recurrence rate is unknown. Here we investigated the relationship between the DFT prior to catheter ablation for peAF and AF recurrence.

**Hypothesis:**

DFT prior to ablation was the predictive factor for AF recurrence after peAF ablation.

**Methods:**

From June 2016 to May 2019, we enrolled 82 consecutive patients (mean age, 65.0 ± 12.4 years), including 45 with peAF and 37 with long‐standing peAF, at Hamamatsu Medical Center. To assess the DFT, we performed IC with gradually increasing energy prior to radiofrequency application.

**Results:**

Forty‐nine and 33 patients showed DFT values less than or equal to 10 J (group A) and greater than 10 J or unsuccessful defibrillation (group B). During the mean follow‐up duration of 20.5 ± 13.1 months, patients in group B showed significantly higher AF recurrence rates than those in group A after the ablation procedure (*p* = .017). Multivariate analysis revealed that DFT was the only predictive factor for AF recurrence (odds ratio, 1.07; 95% CI, 1.00–1.13, *p* = .047).

**Conclusions:**

The DFT for IC was among the strongest prognostic factors in the peAF ablation procedure.

## 1 INTRODUCTION

Catheter ablation is effective for treating drug‐refractory paroxysmal atrial fibrillation (AF).^1^ However, it is less effective for the treatment of persistent AF (peAF) than paroxysmal AF.^2^ Recent reports showed no observable prognosis improvement in patients with peAF after substrate‐targeted catheter ablation as opposed to that observed after pulmonary vein isolation (PVI) alone.^2^ Therefore, to select patients with a good prognosis, it appears necessary to use a new index that correlates with patient prognosis.

AF development is orchestrated by many risk factors, such as high‐blood pressure, overweight/obesity, dyslipidemia, diabetes, tobacco smoking, and excessive drinking.^3^, ^4^, ^5^, ^6^, ^7^, ^8^ Furthermore, risk factors such as the left atrial diameter, left atrial appendage volume, AF duration, left atrial low voltage area, and increased brain natriuretic peptide (BNP) levels contribute to AF recurrence after catheter ablation.^9^, ^10^, ^11^, ^12^, ^13^

Atrial defibrillation threshold (DFT) is correlated with AF duration.^14^, ^15^, ^16^, ^17^ Giuseppo B, et al. indicated that AF duration was a predictor of an increased DFT. Moreover, some studies demonstrated the relationship between the effects of external cardioversion (EC) and catheter ablation.^17^ A high‐energy shock is required for direct‐current EC in peAF; however, internal cardioversion (IC) uses a relatively low‐energy shock but can also be useful for defibrillation. A newly developed IC system with a lower‐energy shock is readily applicable in defibrillation during catheter ablation. This system can eliminate the effects of physique and pulmonary parenchyma on defibrillation, making it possible to precisely estimate a link between AF substrate and DFT. Some studies have demonstrated an association between the DFT for IC and AF recurrence following catheter ablation.^18^, ^19^, ^20^ However, the study designs have different approaches from our study.

In this report, we estimated the DFT for IC prior to catheter ablation for peAF as well as the effects of catheter ablation. Moreover, we investigated whether a high DFT for IC could predict AF recurrence after catheter ablation.

## 2 PATIENTS AND METHODS

### 2.1 Subjects

From June 2016 to May 2019, we enrolled 82 consecutive patients (mean age, 65.0 ± 12.4 years), including 45 with peAF and 37 with long‐standing peAF, at Hamamatsu Medical Center. AF was classified according to the HRS/EHRS/ECAS 2012 Consensus Statement on Catheter and Surgical AF Ablation. PeAF and long‐standing peAF were defined as AF lasting for more than a week and more than a year without resolution, respectively. We excluded patients with prior AF ablation, prior maze procedure, abnormal thyroid function, prior heart valve surgery, ongoing dialysis, or abnormal liver function, and who were lost to follow‐up.

All antiarrhythmic drugs were discontinued at least 1 week prior to the catheter ablation. After the catheter ablation, the medication was continued or discontinued based on the operator's discretion. The medication was occasionally discontinued due to the absence of AF recurrence. All patients were treated with warfarin, dabigatran, rivaroxaban, apixaban, or edoxaban for at least 4 weeks prior to the catheter ablation. The anticoagulant therapy was stopped the morning of the day of the procedure. Intraprocedural anticoagulation for catheter ablation was achieved with heparin at doses that maintained the activated clotting time at >250–300 s. General anesthesia was maintained with propofol.

All patients provided written informed consent before the procedure. The study was conducted in accordance with the Declaration of Helsinki and approved by the hospital's ethics committee.

### 2.2 Internal cardioversion

At the start of the procedure, a Bee AT catheter (Japan Lifeline Co Ltd, Tokyo, Japan) was advanced into the distal coronary sinus (CS) through the right subclavian vein. The distal and middle eight poles were positioned in the distal CS and right atrial lateral wall, respectively. The IC was performed using the Bee AT catheter and a SHOCK AT (Japan Lifeline Co. Ltd., Tokyo, Japan) just before beginning the radiofrequency (RF) application with gradually increasing energy to assess the DFT. The initial IC energy was set to 5 J; thereafter, it was increased according to the following protocol until successful defibrillation was achieved: 5, 10, 15, 20, and 30 J. Patients were divided into two groups based their respective DFT values. We used 10 J as a cut‐off value according to the previous report by Jung W et al.^21^ Patients with DFT values less than or equal to 10 J and greater than 10 J or in whom defibrillation was unsuccessful were divided into groups A and B, respectively.

### 2.3 Catheter ablation procedure

A surface electrocardiogram (ECG) and a bipolar endocardial electrogram were continuously monitored and the ensuing data were stored on a computer‐based digital amplifier (Labsystem Pro, Bard Electrophysiology). After trans‐septal puncture, 10‐polar circular electrode catheters (Lasso, Biosense Webster) and an irrigation catheter (Thermo‐cool, Biosense Webster) were advanced into the left atrium (LA) through the long SL0 sheaths (St. Jude Medical). After the AF cardioversion, the patients underwent circumferential PVI. In all cases, the catheter ablation was completed after confirmation of a conduction block between the pulmonary vein (PV) and the LA. Moreover, a linear ablation was performed at the operator's discretion. A cavotricuspid isthmus ablation was performed at the operator's discretion after the circumferential PVI in patients presenting with atrial flutter on ECG.

### 2.4 Follow‐up

After the catheter ablation, the patients visited our hospital on a monthly basis, during which they underwent an ECG examination, a Holter monitor test (if applicable), or an event recorder check to explore symptoms suggestive of AF recurrence. If these symptoms were found, the patient was instructed to visit our hospital immediately. AF recurrence was defined as a symptomatic and/or documented AF episode lasting more than 30 s.

### 2.5 Statistical analysis

Continuous data are shown as mean ± SD or median and quartile. A logistic regression model was used to determine the predictive factors of a high DFT and recurrent arrhythmia. Survival curves were generated using the Kaplan–Meier technique. For all comparisons, values of *p* < .05 were considered statistically significant. The statistical analysis was performed using MEDCALC version 18 (Medcalc Software Ltd., Ostend, Belgium).

## 3 RESULTS

### 3.1 Patient characteristics

**FIGURE 1:**
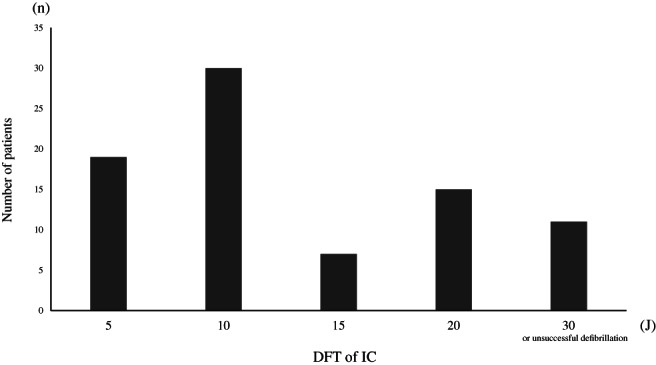
Defibrillation threshold distribution for internal cardioversion

Of the 82 patients, 49 showed DFT values less than or equal to 10 J (5 J, 10 J) (Group A), while 33 showed DFT values greater than 10 J (15 J, 20 J, 30 J, or unsuccessful defibrillation) (Group B). Figure 1 shows the distribution of the DFT for IC as a bar graph. There were no significant intergroup differences in age, body weight, left atrial dimension, ejection fraction, or laboratory findings (Table 1). The ratio of the long‐standing peAF was significantly higher in group B than in group A. In terms of the catheter ablation strategy, a higher ratio of patients in group B underwent box isolation. There was no significant intergroup difference in in the efficacy of antiarrhythmic drug therapy.

**TABLE 1:** Patient characteristics

	GroupA (≦10 J) N = 49	Group B (>10 J) N = 33	*p* value
Patient characteristics			
Male	38 (77.6%)	29 (87.9%)	.24
Age (years)	66.0 ± 12.6	63.3 ± 12.2	.34
Height (cm)	162.2 ± 22.9	167.6 ± 8.33	.2
Body weight (kg)	66.3 ± 14.2	70.2 ± 12.9	.22
Congestive heart failure	9 (18.4%)	8 (24.2%)	.52
Hypertension	12 (24.4%)	9 (27.3%)	.78
Age (>75 years old)	11 (22.4%)	7 (21.2%)	.9
Diabetes mellitus	6 (12.2%)	8 (24.2%)	.16
Stroke	1 (2.0%)	0 (0%)	.41
Laboratory findings			
Hemoglobin (g/dl)	14.5 ± 1.6	14.7 ± 1.3	.43
Total protein (g/dl)	6.81 ± 0.42	6.92 ± 0.40	.25
Albumin (g/dl)	4.05 ± 0.27	4.05 ± 0.28	.98
AST (U/d)	25.9 ± 6.4	25.5 ± 7.0	.84
ALT (U/dl)	21.1 ± 8.3	22.4 ± 14.6	.6
Creatinin (mg/dl)	0.91 ± 0.25	0.92 ± 0.20	.85
BUN (mg/dl)	17.5 ± 6.2	15.8 ± 3.4	.17
HbA1c (%)	6.00 ± 0.67	6.26 ± 1.02	.15
BNP (pg/ml)	132.3 ± 96.9	139.0 ± 121.3	.78
HDL‐C (mg/dl)	56.7 ± 12.4	52.8 ± 13.1	.17
LDL‐C (mg/dl)	109.8 ± 26.2	103.3 ± 20.3	.24
Clinical characteristics			
Long standing PeAF (>1 year)	16 (32.7%)	21(63.4%)	<.01
Left atrial dimension(mm)	41.7 ± 6.1	43.4 ± 4.0	.15
Ejection fraction (%)	59.3 ± 10.1	57.0 ± 12.3	.35
Ablation strategy			
PVI	49 (100%)	33 (100%)	N/A
CTI block	11(22.4%)	5 (15.2%)	.42
Roof line	6 (12.2%)	4 (12.1%)	.99
SVC isolation	4 (8.1%)	3 (9.1%)	.88
BOX isolation	7 (14.3%)	16 (48.5%)	<.01
Antiarrhythmic drugs			
Class Ic	12 (24.5%)	7 (21.2%)	.73
Sotalol	0 (0%)	1 (3.0%)	.23
Amiodarone	0 (0%)	0 (0%)	N/A
Beprizil	8 (16.3%)	9 (27.3%)	.24

*Note*: Values are the mean ± SD or n (%). BOX isolation: isolation of the posterior LA, including all PVs.

Abbreviations: BNP, B‐type natriuretic peptide; CTI, cavotricuspid isthmus; PVI, pulmonary vein isolation.

### 3.2 AF recurrence during follow‐up

**FIGURE 2:**
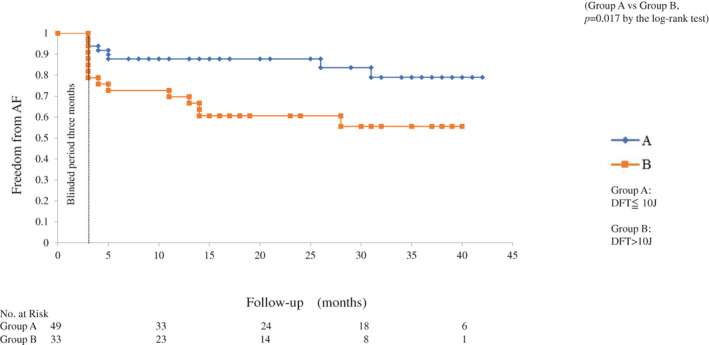
Kaplan–Meier curve analysis of the incidence of atrial fibrillation recurrence after the ablation procedure. Group A: equal or lower than 10 J (5 J, 10 J). Group B: higher than 10 J or unsuccessful defibrillation (15 J, 20 J, 30 J, or unsuccessful defibrillation)

In group A, AF recurrence rates were 6.2%, 12.3%, and 21.1%, 3 months, 1 year, and 3 years post‐ablation, respectively. In group B, AF recurrence rates were 21.2%, 33.3%, and 44.5%, 3 months, 1 year, and 3 years post‐ablation, respectively. During the mean follow‐up duration of 20.5 ± 13.1 months, group B patients showed a significantly higher AF recurrence rate than group A patients after the catheter ablation (*p* = .017) (Figure 2).

### 3.3 Complications

A patient in group B developed pericardial tamponade, which resolved after pericardial drainage. Barring the aforementioned complication, no other major complications such as symptomatic PV stenosis, cerebral embolism, phrenic nerve palsy, or atrioesophageal fistula were noted.

### 3.4 Patient characteristics of AF recurrence versus AF non‐recurrence group

The ratios of the long‐standing peAF and DFT were significantly higher in the AF recurrence than that of the AF non‐recurrence group (Table 2).

**TABLE 2:** Patient characteristics of AF reccurence group versus AF non‐reccurence group

	AF reccurence group N = 22	AF non‐reccurence group N = 60	*p* value
Patient characteristics			
Male	19 (86.3%)	48 (80.0%)	.51
Height (cm)	160.8 ± 33.4	165.7 ± 8.3	.29
Body weight (kg)	70.7 ± 18.7	66.8 ± 11.4	.26
Age (>75 years old)	6 (27.3%)	15 (25.0%)	.84
Laboratory findings			
Hemoglobin (g/dl)	14.5 ± 1.7	14.6 ± 1.4	.78
Total protein (g/dl)	6.9 ± 0.4	6.9 ± 0.4	.79
Albumin (g/dl)	4.1 ± 0.4	4.0 ± 0.2	.74
AST (U/d)	26.0 ± 6.9	25.6 ± 6.5	.80
ALT (U/dl)	25.2 ± 15.7	20.3 ± 8.8	.08
Creatinin (mg/dl)	1.0 ± 0.3	0.9 ± 0.2	.20
BUN (mg/dl)	16.8 ± 6.6	16.8 ± 4.7	1.00
HbA1c (%)	6.3 ± 1.2	6.0 ± 0.7	.11
BNP (pg/ml)	123.6 ± 126.7	139.2 ± 99.2	.56
HDL‐C (mg/dl)	54.7 ± 14.3	55.4 ± 12.3	.86
LDL‐C (mg/dl)	110.2 ± 20.0	106.1 ± 25.5	.50
Clinical characteristics			
Long standing PeAF (>1 year)	14 (63.6%)	23 (38.3%)	.042
Left atrial dimension(mm)	42.4 ± 4.5	42.4 ± 5.7	.99
Ejection fraction (%)	59.8 ± 10.3	57.9 ± 11.3	.49
DFT (J)	17.5 ± 8.6	12.1 ± 7.8	.0079
Ablation strategy			
PVI	22 (100%)	60(100%)	N/A
CTI block	5 (22.7%)	12(20.0%)	.79
Roof line	1 (4.5%)	8(13.3%)	.26
SVC isolation	2 (9.0%)	5 (8.3%)	.91
BOX isolation	7 (31.8%)	16 (26.7%)	.65
Antiarrhythmic drugs			
Class Ic	4 (18.2%)	15 (25.0%)	.52
Sotalol	1 (4.5%)	0 (0%)	.10
Amiodarone	0 (0%)	0 (0%)	N/A
Beprizil	5 (22.7%)	12 (20.0%)	.79

*Note*: Values are the mean ± SD or n (%). BOX isolation: isolation of the posterior LA, including all PVs.

Abbreviations: BNP, B‐type natriuretic peptide; CTI, cavotricuspid isthmus; PVI, pulmonary vein isolation.

### 3.5 Predictors of AF recurrence

Table 3 shows post‐procedural ablation predictors of AF recurrence after univariate and multivariate analyses. Univariate analysis revealed that long‐standing peAF (odds ratio [OR], 2.82; 95% CI, 1.02–7.74; *p* = .041) and the DFT (OR, 1.08; 95% CI, 1.02–1.15; *p* < .001) were the predictive factors for AF recurrence. However, multivariate analysis revealed that DFT was the only predictive factor for AF recurrence (OR, 1.07; 95% CI, 1.00–1.13; *p* = .047).

**TABLE 3:** Predictors of recurrent arrhythmia after the ablation procedure on univariate analysis and multivariate analysis

Variables	Univariate analysis OR (95% CI)	*p* value	Multivariate analysis OR (95% CI)	*p* value
Patient characteristics				
Male	1.58 (0.40–6.24)	.5		
Height (cm)	0.99 (0.96–1.01)	.32		
Body weight (kg)	1.02 (0.99–1.06)	.26		
Age (>75 years old)	1.13 (0.37–3.40)	.84		
Laboratory findings				
Hemoglobin (g/dl)	0.95 (0.68–1.33)	.77		
Total protein (g/dl)	1.17 (0.36–3.83)	.79		
Albumin (g/dl)	1.36 (0.22–8.51)	.74		
AST (U/dl)	1.00 (0.94–1.09)	.79		
ALT (U/dl)	1.04 (0.99–1.08)	.09		
Creatinin (mg/dl)	3.97 (0.47–33.19)	.2		
BUN (mg/dl)	1.00 (0.91–1.10)	.99		
HbA1c (%)	1.55 (0.88–2.71)	.12		
BNP (pg/ml)	1.00 (0.99–1.00)	.54		
HDL‐C (mg/dl)	1.00 (0.96–1.04)	.86		
LDL‐C (mg/dl)	1.01 (0.99–1.03)	.49		
Clinical characteristics				
Long standing PeAF (>1 year)	2.82 (1.02–7.74)	.041	1.97 (0.66–5.87)	.22
Left atrial dimension (mm)	0.99 (0.91–1.10)	.99		
Ejection fraction (%)	1.02 (0.97–1.07)	.48		
DFT (J)	1.08 (1.02–1.15)	<.01	1.07 (1.00–1.13)	.047
Ablation strategy				
CTI block	1.18 (0.36–3.83)	.79		
Roof line	0.30 (0.036–2.63)	.22		
SVC isolation	1.10 (0.19–6.13)	.91		
BOX isolation	1.28 (0.44–3.72)	.65		
Antiarrhythmic drugs				
Class Ic	0.57 (0.15–2.23)	.40		
Beprizil	3.23 (0.84–12.50)	.09		

Abbreviations: CI, confidence interval; OR, odds ratio.

## 4 DISCUSSION

In this study, we investigated an association between the DFT for IC and AF recurrence after peAF ablation. In patients with peAF, the AF recurrence rate increased with the DFT for IC despite the addition of RF applications after PVI (such as box isolation). Moreover, DFT for IC is among the strongest predictors of AF recurrence.

In our study, approximately 10 J was considered the average DFT for peAF and set as the cut‐off value as a review article^21^ reported that the average DFT for IC of 25 patients with peAF was 9.1 ± 7.4 J. Another review^22^ reported a difference in DFT between paroxysmal and peAF.

Recent reports demonstrated that catheter ablation can be an effective treatment for peAF to maintain the sinus rhythm. Furthermore, factors contributing to the success rates of catheter ablation for AF have been studied.^10^, ^11^ Liu et al. reported that the LA diameter contributes to the prognosis of patients with AF after catheter ablation.^10^ Moreover, Pinto et al. reported that the LA appendage volume contributes to AF recurrence.^11^ In contrast, Masuda et al.^12^ reported that the LA diameter and AF recurrence are not correlated, and whether they are related remains controversial. Our study did not confirm the relationship between the LA diameter and AF recurrence. Considerable LA enlargement might not have been an indication for ablation, which could have resulted in selection bias. Zyng Y et al. described that high‐BNP levels were associated with AF recurrence after catheter ablation.^13^ In contrast, our study found no relationship between the BNP level and AF recurrence. This might be attributed to the policy of our institute, where ablation of AF is performed after adequate treatment for heart failure. The relationship between the BNP level and AF recurrence after catheter ablation requires further investigation in meta‐analyses.

Some studies have reported AF recurrence after IC without ablation. Biffi M et al. showed that AF recurrence after IC occurred more frequently in patients in whom AF evolved continuously for more than 3 years.^14^ Giuseppo B et al. indicated that AF duration was the strongest predictor of increased DFT.^15^ Furthermore, another study reported that AF recurrence after IC occurred more frequently in patients with chronic AF and left ventricular dysfunction (ejection fraction <40%).^16^ However, a few studies reported a correlation between the DFT values and the prognosis of patients with AF after catheter ablation. Kim et al. showed that a high DFT for EC was associated with an increased risk of AF recurrence in patients with long‐standing peAF.^17^ Nevertheless, the study designs differed from that of our study. For example, we performed IC instead of EC. The high‐energy shock used in EC is comparatively higher than that used in IC; consequently, the impact of the shock energy on the patient is greater. IC may be a promising mainstream technique for defibrillation in AF ablation. We also analyzed the AF recurrence rate and DFT in patients with long‐standing peAF as well as in patients with up to a 1‐year history of AF. Ihara et al. reported that a high DFT for IC indicated arrhythmogenicity of the superior vena cava in non‐long‐standing peAF.^18^ Komatsu et al. reported that, among a total of 128 patients with peAF, if AF did not terminate during ablation, a 5‐J IC protocol was started and increased incrementally in 5‐J steps until successful cardioversion was accomplished. When AF does not terminate after the completion of the predetermined stepwise ablation, further extensive ablation to terminate AF might be unnecessary if the AF can be successfully terminated by electrical cardioversion at low DFT.^19^ In our study, IC was performed just before starting the catheter ablation, which differs from this report. This study differs from that of Yao et al.^20^ with its DFT cut‐off of 5 J and in its procedural technique.

Our study showed that a high DFT for IC before RF ablation predisposed patients to a poor prognosis of AF after catheter ablation. The box isolation rate was significantly higher in group B than in group A patients. Box isolation might have been performed in many intractable cases; however, no significant intergroup differences were observed in the ablation strategy. Complete PVI was achieved in both of our study groups. Moreover, it is assumed that the ablation strategy did not affect AF recurrence in either of our study groups. In group B, with a high DFT, the percentage of patients with long‐standing peAF was significantly higher than that in group A, which probably influenced AF prognosis after ablation.

Our study demonstrated that a high DFT was the strongest prognostic factor for AF recurrence after the ablation procedure. Therefore, we speculate that a high DFT is correlated with atrial remodeling, due to which the success rate of catheter ablation in AF is gradually decreasing. The concept of the left atrial low‐voltage areas is a well‐established marker for atrial remodeling and AF recurrence after PVI.^12^ The presence of left atrial low‐voltage areas may correlate with the high DFT; however, this was not investigated in this study.

In recent reports, in addition to PV isolation, additional ablation targeting the left atrial substrate using linear ablation,^23^ CFAE ablation,^24^ CARTOFINDER,^25^ and ExTRa mapping^26^ is reportedly effective for improving the prognosis of peAF ablation. To improve the performance after peAF ablation in patients with a high DFT, these methods may reduce recurrence after AF ablation.

## 5 LIMITATION

This study has some limitations that should be noted. First, catheter position may be correlated with the DFT. However, in some cases, the Bee AT catheter could not be positioned properly due to right atrial enlargement, which possibly affected the DFT. As such, there may be problems with the reproducibility of the DFT. Second, we found no correlation between the LA diameter and AF recurrence. Only patients who underwent ablation were included; thus, selection bias may have occurred at the indicated stage, resulting in no correlation between the LA diameter and AF recurrence. Hence, a randomized study with a larger study population is needed to clarify the relationship between the DFT and AF prognosis after catheter ablation.

## 6 CONCLUSION

In patients with peAF undergoing IC with a high DFT, the AF recurrence rate was higher even with additional RF applications after PVI (such as box isolation). Moreover, DFT for IC is among the strongest predictors for AF recurrence.

## Supporting information

**Appendix** S1: Supporting information

## Data Availability

Data of this research are not shared.
