# Supplementary figures and images for: Defibrillation threshold of internal cardioversion prior to ablation predicts atrial fibrillation recurrence
Source: Clin Cardiol. 2021 Jun 23;44(8):1169–76. doi: 10.1002/clc.23679 (PMC8364735; doi:10.1002/clc.23679)

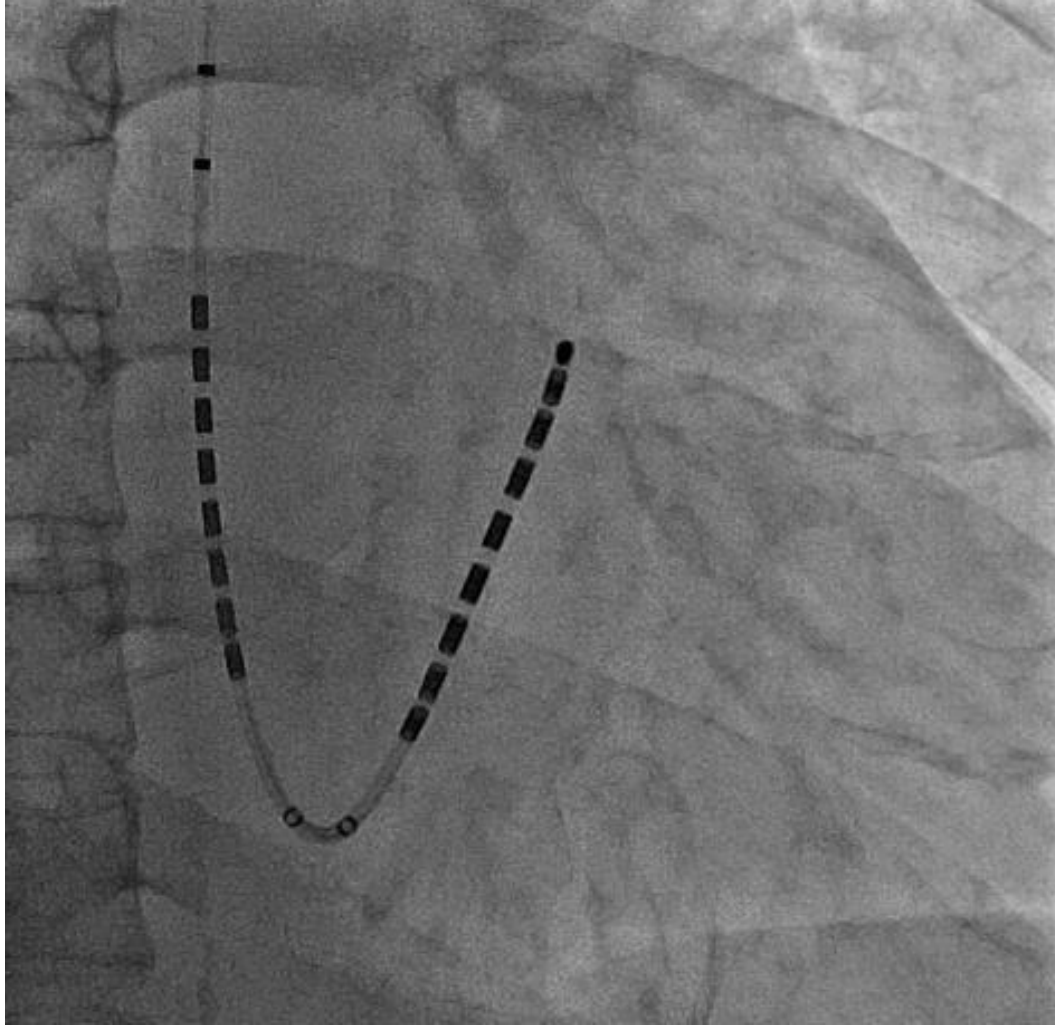

RAO30

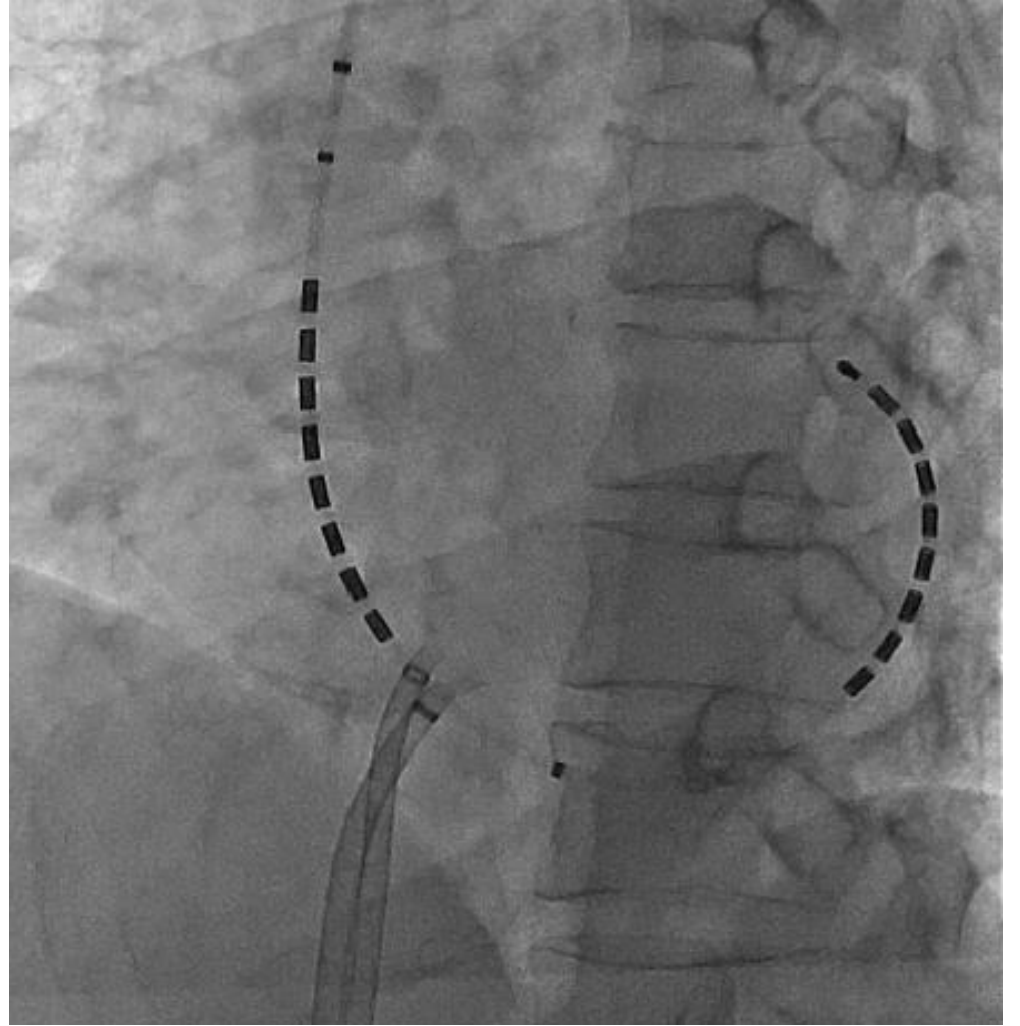

LAO50

Supplement: Supplementary file 1 — Appendix S1: Supporting information [file CLC-44-1169-s001.pdf]
